# Possible Association of Polymorphisms in Ubiquitin Specific Peptidase 46 Gene With Post-traumatic Stress Disorder

**DOI:** 10.3389/fpsyt.2021.663647

**Published:** 2021-08-11

**Authors:** Jun Ho Seo, Tae Yong Kim, Se Joo Kim, Jin Hee Choi, Hyung Seok So, Jee In Kang

**Affiliations:** ^1^Institute of Behavioral Science in Medicine and Department of Psychiatry, Yonsei University College of Medicine, Seoul, South Korea; ^2^Department of Neuropsychiatry, Veterans Health Service Medical Center, Seoul, South Korea

**Keywords:** post-traumatic stress disorder, ubiquitin proteasome system, ubiquitin specific peptidase, USP46, genetic association study

## Abstract

**Introduction:** Dynamic proteolysis, through the ubiquitin-proteasome system, has an important role in DNA transcription and cell cycle, and is considered to modulate cell stress response and synaptic plasticity. We investigated whether genetic variants in the ubiquitin carboxyl-terminal hydrolase 46 (*USP46*) would be associated with post-traumatic stress disorder (PTSD) in people with exposure to combat trauma using a case-control candidate gene association design.

**Methods:** Korean male veterans exposed to the Vietnam War were grouped into those with (*n* = 128) and without (*n* = 128) PTSD. Seven tagging SNPs of *USP46* were selected, and single-marker and haplotype-based association analyses were performed. All analyses were adjusted for sociodemographic factors and levels of combat exposure severity and alcohol problem.

**Results:** One single-marker (rs2244291) showed nominal evidence of association with PTSD status and with the “re-experiencing” cluster, although the association was not significant after Bonferroni correction. No significant association with the other SNPs or the haplotypes was detected.

**Conclusion:** The present finding suggests preliminarily that genetic vulnerability regarding the ubiquitin-proteasome system may be related to fear memory processes and the development of PTSD symptoms after trauma exposure. Further studies with a larger sample size will be needed to examine the role of the ubiquitin-proteasome system including USP46 in PTSD.

## Introduction

Post-traumatic stress disorder (PTSD) is a chronic and debilitating condition with characteristic symptoms, including re-experience of fear memory and severe anxiety as long-term responses to life-threatening traumatic exposure (1). However, not all people who are exposed to trauma develop PTSD. For instance, only around 10–20% of veterans exposed to combat trauma develop PTSD (2–4). The reason why certain individuals are more likely to develop PTSD than others after similar trauma exposure has not been elucidated. The molecular determinants of individual differences in vulnerability or resilience to stressors are still not well-understood. Twin studies have shown that PTSD is moderately heritable, with approximately 40% of the variance in PTSD attributable to genetic variance (5–7). Accummulating evidence shows that genetic factors contribute to the PTSD susceptibility among people who have experienced trauma (8, 9).

There is emerging literature on genetic variations in mechanisms responsible for development and maintenance of PTSD (9–11). Many studies on detection of candidate genes associated with PTSD have focused on genetic variations of the dopaminergic and serotonergic systems (12–15), but robust common genetic variants have yet to be identified. Since ubiquitin-proteasome-mediated proteolysis plays a crucial role in synaptic development and long-term synaptic plasticity in neural circuits (16–20), the ubiquitin-proteasome system is an interesting biological target for the pathophysiology of human neuropsychiatric disorders related to stress-related synaptic plasticity, such as PTSD. Dynamic and reversible processes via the ubiquitin-proteasome system regulate synaptic Alpha-amino-3-hydroxy-5-Methyl-4-isoxazolepropionic acid—type glutamate receptors (AMPARs) levels, which is known to be important for controlling development and function of glutamatergic synapses and long-term potentiation of synaptic transmission in the brain (21, 22). Substantial evidence from animal studies suggests that ubiquitin-mediated proteolysis is an important regulation process for fear memory formation and reconsolidation (23–26). To date, little is known about the genetic evidence of the role of ubiquitin-proteasome system in clinical samples of individuals with PTSD.

Ubiquitin carboxyl-terminal hydrolase 46 (USP46), a deubiquitinating enzyme that is widely expressed throughout the brain (19), was identified in *Caenorhabditis elegans* as the first deubiquitinating enzyme to regulate degradation of glutamate receptors and reported to have several roles in the nervous system (27). In mammalian studies, both *in vivo* and *in vitro*, USP46 has been implicated in regulating the AMPA glutamatergic system (28, 29), which is important for inter-neuronal communication and higher brain functions such as learning and memory. For example, altered AMPAR expression has been shown to modulate contextual fear memory reconsolidation (30, 31) and endocytosis of AMPARs has been demonstrated to be required for the loss of fear response during adaptive reconsolidation of contextual fear (32). In addition, USP46 has been implicated in regulating the GABAergic system (33–36), which also has a crucial role in fear memory formation, reconsolidation, and extinction (37–42). Notably, Ebihara and colleagues reported that *Usp46* knockout mice display shortened immobility times in the tail suspension test (36) and long-term memory deficits in the object recognition test (33). They also found out that *Usp46* mutant mice were more sensitive to stress and developed impaired maternal behaviors (43). The findings on the possible regulatory role of the USP46 in synaptic plasticity and fear memory processing suggest that USP46 may be an interesting candidate for the development and recovery of fear-related disorders such as PTSD. Although *USP46* single nucleotide polymorphism (SNP) has been reported to be associated with major depressive disorder (44) and with depressive temperament (45) in human, no study has been conducted to examine the association of the *USP46* gene with PTSD in a clinical sample.

We investigated whether the genetic variants of the *USP46* would be associated with chronic PTSD status in Korean male veterans with exposure to combat trauma using a case-control candidate gene association design. Our main hypothesis was that susceptibility to PTSD might be associated with genetic polymorphisms of the *USP46*.

## Materials and Methods

### Participants and Procedure

According to the DSM-IV-TR diagnostic criteria (1) for PTSD, 128 subjects with PTSD and 128 (non-PTSD) controls were recruited from a psychiatric outpatient clinic at the Veterans Health Service (VHS) Medical Center. All subjects were of Korean ethnicity and male veterans who had served on active duty during the Vietnam War. Individuals with a history of head trauma, organic brain syndrome including cerebrovascular accidents or dementia, major psychiatric disorders including psychosis or bipolar disorder, or substance dependence other than alcohol and nicotine were excluded. The study was approved by the institutional review board of the VHS Medical Center, South Korea (BOHUN 2016-02-007). All subjects gave their written informed consent before participating in this study.

### Measures

For assessing PTSD, we used the Clinician-Administered PTSD Scale (CAPS), a structured clinical interview, which is considered the gold standard for diagnosing PTSD (46, 47). The diagnosis of PTSD was determined by symptom frequency and intensity based on the liberal scoring rule of the CAPS (48). In addition, the Combat Exposure Scale (CES), a self-reporting scale, was administered for measuring the level of wartime traumatic stressors experienced by the combatants (49). The total CES scores were divided into five categories of combat exposure: light (1–8), light–moderate (9–16), moderate (17–24), moderate–heavy (25–32), and heavy (33–41). The Alcohol Use Disorders Identification Test (AUDIT) was also used to assess hazardous and harmful alcohol use (50).

### Genotyping

Seven tagging SNPs covering all regions of *USP46* (rs346005, rs10034164, rs2244291, rs12646800, rs6554557, rs17675844, and rs10517263) were selected with the criteria of an *r*^2^ threshold >0.8 based on a prior genetic association study in a Japanese population in which gene-based approach was used involving all common SNPs (minor allele frequency >5%) (44). Subjects donated a blood sample through venipuncture, and the DNA of each subject was isolated using extraction protocol with QG-810/800 of Quickgene DNA whole blood kit-S after lysate preparation. The genotyping procedures were carried out using single base primer extension assay using the ABI PRISM SNaPShot multiplex kit (ABI, Foster City, CA, USA) according to the manufacturer's recommendations. The forward and reverse primer pairs used for the SNaPshot assay and genetic information for all tested SNPs are presented in Supplementary Table 1. Analysis was performed using Genemapper software (version 3.0; Applied Biosystems) in the DNA Link, Inc. (Seoul, South Korea).

### Data Analyses

Demographic and clinical characteristics between subjects with and without PTSD were compared using χ^2^-test or Student's *t*-test on the Statistical Package for the Social Sciences version 25.0 (SPSS Inc., Chicago, IL, USA). The Hardy–Weinberg equilibrium for each SNP in the control group was calculated by χ^2^-test. Statistical power was calculated with Genetic Association Study Power Calculator (https://csg.sph.umich.edu/abecasis/gas_power_calculator). Given the available sample size, the statistical power for detecting a risk allele with an effect size of 1.6 is 88%, depending on 10% minor allele frequency, 40% lifetime disease prevalence of war veterans, and 5% alpha level.

Single-marker analyses were performed using the R package SNPassoc (51). Between-group comparisons of genotype frequency differences for diagnostic status were performed by logistic regression analysis considering different genetic inheritance models. For five SNPs whose genotype frequencies of homozygous with minor alleles were <5%, dominant genetic model was assumed. The outcome variable was analyzed yielding odds ratios (ORs) with 95% confidence intervals (CIs) and *p*-values. Associations between haplotype distributions and PTSD status were examined using the “haplo.score” function of R package haplo.stats (52). This package computes score statistics to test associations between haplotypes and a trait allowing adjustment for other determinants. This analysis was corrected for multiple testing by applying the simulate = TRUE parameter in haplo.score which gives simulated *p*-values. These simulated haplotype score statistics are calculated from a permuted re-ordering of the trait (PTSD status) and *USP46* polymorphisms. We used 100,000 permutations for all the analyses. Haploblock structure and linkage disequilibrium (LD) patterns obtained from the seven SNPs were constructed using the Haploview ver.4.2 (http://www.broad.mit.edu/mpg/haploview). In further analysis, we conducted linear regression analyses for three clusters (re-experiencing, avoidance, and hyperarousal) of PTSD symptoms considering PTSD as continuous phenotypes. Demographic and clinical characteristics which were different between cases and controls in χ^2^-test or *t*-test (*p* < 0.1) or which have been reported as a risk factor of PTSD in previous studies (53, 54) were selected as potential confounders in analyses. As a result, all analyses were adjusted for demographic factors including age, education year, socio-economic status, and marital status; the five levels of CES, and AUDIT scores (harmful alcohol drinking). In all analyses, *p*-value of <0.05 was considered as nominally significant (uncorrected *p* < 0.05). The statistical threshold was corrected using the Bonferroni method for the total number of SNPs (α = 0.05/7 = 0.0071).

## Results

The demographic and clinical characteristics of subjects with and without PTSD are presented in Table 1. The groups with and without PTSD were not significantly different in terms of age, education level, marital status, and socioeconomic status. For combat exposure, the distribution of the five CES categories showed a significant difference between PTSD and non-PTSD groups (χ^2^ = 48.54, *df* = 4, *p* < 0.001), with a higher proportion of heavy trauma experience in subjects with PTSD than those without PTSD. For alcohol problem, subjects with PTSD had significantly harmful alcohol consumption based on the AUDIT score, compared to those without PTSD (11.66 ± 10.92 vs. 6.84 ± 7.53, *p* < 0.001).

**Table 1:** Demographic and clinical characteristics of the study participants.

	**Non-PTSD** **(*N* = 128)**	**PTSD** **(*N* = 128)**	***T* or χ^**2**^**	***P-value***
Age	62.92 ± 4.32	63.15 ± 3.55	0.46	0.647
Education (years)	10.53 ± 3.12	10.38 ± 2.83	−0.40	0.690
Marital status:				
Married/others, *n*	119/9	110/18	3.35	0.067
Socioeconomic status:				
Low/Medium/High, *n*	45/63/20	44/59/25	0.70	0.705
Combat exposure:				
Light/Light-moderate/Moderate/Moderate-heavy/Heavy, *n*	38/48/30/11/1	6/29/60/26/7	48.54	<0.001
AUDIT score	6.84 ± 7.53	11.66 ± 10.92	4.11	<0.001
Total CAPS	8.84 ± 11.39	62.85 ± 22.27	24.42	<0.001
Re-experiencing	4.16 ± 7.38	20.72 ± 8.56	16.58	0.001
Avoidance	1.57 ± 3.34	21.46 ± 10.18	21.00	<0.001
Hyperarousal	3.19 ± 4.18	20.67 ± 8.15	17.48	<0.001

The allelic distributions of the seven SNPs in the control group were in accordance with the Hardy–Weinberg equilibrium (Supplementary Table 2). In single-marker analyses under multiple genetic models, only one single-marker (rs2244291) showed a significant association at the nominal significance level of 5% (*p* = 0.0193 in over-dominant model and *p* = 0.0497 in Co-dominant model), but the association did not remain significant after stringent correction for multiple comparisons (Table 2). For the other SNPs in the *USP46* region, no significant association was found between the groups (Table 2).

**Table 2:** Association of the *USP46* SNPs under different genetic models with PTSD status.

**SNP**	**Model**	**Genotype**	**Non-PTSD**	**PTSD**	**OR(95% CI)**	***P*-value**	**AIC**
rs346005	Co-dominant	A/A	42 (35.9%)	35 (29.9%)	1.00	0.4781	279.1
		A/C	49 (41.9%)	61 (52.1%)	1.33 (0.67–2.65)		
		C/C	26 (22.2%)	21 (17.9%)	0.84 (0.36–1.96)		
	Dominant	A/A	42 (35.9%)	35 (29.9%)	1.00	0.6570	278.4
		A/C–C/C	75 (64.1%)	82 (70.1%)	1.16 (0.61–2.19)		
	Recessive	A/A–A/C	91 (77.8%)	96 (82.1%)	1.00	0.3719	277.8
		C/C	26 (22.2%)	21 (17.9%)	0.71 (0.34–1.50)		
	Over-dominant	A/A–C/C	68 (58.1%)	56 (47.9%)	1.00	0.2504	277.3
		A/C	49 (41.9%)	61 (52.1%)	1.42 (0.78–2.60)		
	Log-additive	–	117 (50.0%)	117 (50.0%)	0.96 (0.63–1.45)	0.8312	278.6
rs10034164	Co-dominant	T/T	85 (71.4%)	87 (73.7%)	1.00	0.3382	283.2
		C/T	31 (26.1%)	26 (22.0%)	0.59 (0.29–1.21)		
		C/C	3 (2.5%)	5 (4.2%)	1.05 (0.21–5.18)		
	Dominant	T/T	85 (71.4%)	87 (73.7%)	1.00	0.1926	281.7
		C/T–C/C	34 (28.6%)	31 (26.3%)	0.64 (0.32–1.26)		
	Recessive	T/T–C/T	116 (97.5%)	113 (95.8%)	1.00	0.8017	283.4
		C/C	3 (2.5%)	5 (4.2%)	1.22 (0.25–5.90)		
	Over-dominant	T/T–C/C	88 (73.9%)	92 (78.0%)	1.00	0.1412	281.3
		C/T	31 (26.1%)	26 (22.0%)	0.59 (0.29–1.20)		
	Log-additive	–	119 (50.2%)	118 (49.8%)	0.75 (0.43–1.32)	0.3227	282.4
rs2244291	Co-dominant	A/A	86 (67.2%)	73 (57.0%)	1.00	^*^0.0497	307.1
		A/G	33 (25.8%)	50 (39.1%)	1.95 (1.06–3.59)		
		G/G	9 (7.0%)	5 (3.9%)	0.61 (0.16–2.32)		
	Dominant	A/A	86 (67.2%)	73 (57.0%)	1.00	0.0765	308.0
		A/G–G/G	42 (32.8%)	55 (43.0%)	1.68 (0.94–3.00)		
	Recessive	A/A–A/G	119 (93.0%)	123 (96.1%)	1.00	0.2613	309.9
		G/G	9 (7.0%)	5 (3.9%)	0.48 (0.13–1.77)		
	Over-dominant	A/A–G/G	95 (74.2%)	78 (60.9%)	1.00	^*^0.0193	305.6
		A/G	33 (25.8%)	50 (39.1%)	2.03 (1.11–3.69)		
	Log-additive	–	128 (50.0%)	128 (50.0%)	1.29 (0.80–2.07)	0.2952	310.0
rs12646800	Co-dominant	C/C	107 (89.9%)	102 (86.4%)	1.00	0.7025	283.3
		C/T	12 (10.1%)	16 (13.6%)	1.20 (0.47–3.10)		
	Log-additive	–	119 (50.2%)	118 (49.8%)	1.20 (0.47–3.10)	0.7025	283.3
rs6554557	Co-dominant	A/A	85 (71.4%)	86 (73.5%)	1.00	0.3471	281.3
		A/C	31 (26.1%)	27 (23.1%)	0.59 (0.29–1.22)		
		C/C	3 (2.5%)	4 (3.4%)	0.69 (0.13–3.69)		
	Dominant	A/A	85 (71.4%)	86 (73.5%)	1.00	0.1484	279.4
		A/C–C/C	34 (28.6%)	31 (26.5%)	0.60 (0.30–1.20)		
	Recessive	A/A–A/C	116 (97.5%)	113 (96.6%)	1.00	0.8020	281.4
		C/C	3 (2.5%)	4 (3.4%)	0.81 (0.15–4.24)		
	Over-dominant	A/A–C/C	88 (73.9%)	90 (76.9%)	1.00	0.1650	279.5
		A/C	31 (26.1%)	27 (23.1%)	0.61 (0.30–1.24)		
	Log-additive	–	119 (50.4%)	117 (49.6%)	0.68 (0.38–1.21)	0.1911	279.7
rs17675844	Co-dominant	A/A	101 (86.3%)	94 (81.7%)	1.00	0.2047	276.3
		C/A	15 (12.8%)	20 (17.4%)	2.13 (0.92–4.91)		
		C/C	1 (0.9%)	1 (0.9%)	1.02 (0.03–35.28)		
	Dominant	A/A	101 (86.3%)	94 (81.7%)	1.00	0.0825	274.5
		C/A–C/C	16 (13.7%)	21 (18.3%)	2.05 (0.91–4.64)		
	Recessive	A/A–C/A	116 (99.1%)	114 (99.1%)	1.00	0.9888	277.5
		C/C	1 (0.9%)	1 (0.9%)	0.98 (0.03–33.44)		
	Over-dominant	A/A–C/C	102 (87.2%)	95 (82.6%)	1.00	0.0749	274.3
		C/A	15 (12.8%)	20 (17.4%)	2.08 (0.92–4.91)		
	Log-additive	–	117 (50.4%)	115 (49.6%)	1.86 (0.87–4.00)	0.1061	274.9
rs10517263	Co-dominant	C/C	93 (78.8%)	97 (82.2%)	1.00	0.3338	281.7
		G/C	24 (20.3%)	20 (16.9%)	0.56 (0.25–1.23)		
		G/G	1 (0.8%)	1 (0.8%)	0.65 (0.04–11.49)		
	Dominant	C/C	93 (78.8%)	97 (82.2%)	1.00	0.1394	279.7
		G/C–G/G	25 (21.2%)	21 (17.8%)	0.56 (0.26–1.22)		
	Recessive	C/C–G/C	117 (99.2%)	117 (99.2%)	1.00	0.8291	281.8
		G/G	1 (0.8%)	1 (0.8%)	0.73 (0.04–12.88)		
	Over-dominant	C/C–G/G	94 (79.7%)	98 (83.1%)	1.00	0.1467	279.8
		G/C	24 (20.3%)	20 (16.9%)	0.56 (0.25–1.23)		
	Log-additive	–	118 (50.0%)	118 (50.0%)	0.60 (0.30–1.23)	0.1579	279.9

*OR, odds ratio; CI, confidence interval; AIC, akaike information criterion of each genetic model*.

* *p < 0.05*.

**Figure 1:**
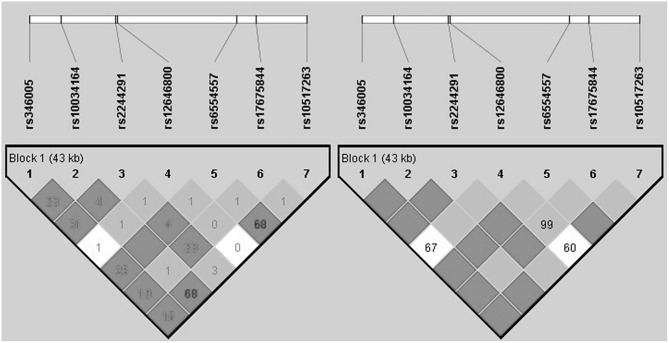
Haploblock structure and linkage disequilibrium for the non-PTSD group from tagging SNPs of *USP46*. The color scheme is based on *r*^2^-value (left) and D prime value (right).

The analysis of LD and haplotype block for the *USP46* revealed one haplotype block (Figure 1). In haplotype analyses, the permutation test of the seven SNP haplotypes showed no significant difference in the estimated haplotype frequency distributions between both groups (Table 3).

**Table 3:** The effects of *USP46* Haplotype on the affected status of PTSD.

**Block 1^a^**							**Hap-Freq^b^**	**Hap-Score^c^**	**Crude *p*-val^d^**	**Sim. *p*-val^e^**
**rs346005**	**rs10034164**	**rs2244291**	**rs12646800**	**rs6554557**	**rs17675844**	**rs10517263**				
C	C	A	C	C	A	G	0.0997	−1.3480	0.1777	0.1856
C	T	A	C	A	A	C	0.0718	−1.1957	0.2318	0.2389
C	T	G	C	A	A	C	0.1284	0.1278	0.8983	0.9007
A	T	A	C	A	A	C	0.5052	0.2355	0.8138	0.8148
C	C	A	C	C	A	C	0.0514	0.3377	0.7356	0.7417
A	T	A	T	A	A	C	0.0521	0.3821	0.7024	0.7084
C	T	G	C	A	C	C	0.0845	1.3005	0.1934	0.1996

a *Global-stat = 4.91724, df = 7, p = 0.67006, global simulated p = 0.69839*.

b *Hap-Freq, estimated frequency of the haplotype in the pool of all participants*.

c *Hap-Score, score for the haplotype*.

d *Asymptotic chi-square p-value*.

e *Simulated p-value*.

In further analyses considering PTSD as continuous phenotypes, the rs2244291, which was shown to be nominally significantly associated with PTSD status in the main analysis, was associated with the “re-experiencing” cluster of PTSD symptoms (*p* = 0.014 in over-dominant model and *p*= 0.041 in co-dominant model) (Table 4).

**Table 4:** The effects of *USP46* rs2244291 on three clusters of PTSD symptom.

**Cluster**	**Model**	**Genotype**	***n***	**Mean (S.E)**	**Mean difference** **(95% CI)**	***p*-value**	**AIC**
Re-experiencing		A/A	159	11.56(0.09)			
	Co-dominant	A/G	83	14.71(1.32)	3.10(0.50 to 5.70)	0.041^*^	1902
		G/G	14	8.93(2.90)	−1.54(−6.91 to 3.83)		
	Dominant	A/A	159	11.56(0.89)		0.054	1902
		A/G–G/G	97	13.88(1.21)	2.46(−0.03 to 4.95)		
	Recessive	A/A–A/G	242	12.64(0.74)		0.326	1905
		G/G	14	8.93(2.90)	−2.68(−8.02 to 2.66)		
	Over-dominant	A/A–G/G	173	11.35(0.85)		0.014^*^	1900
		A/G	83	14.71(1.32)	3.24(0.68 to 5.79)		
	Log-additive	–	–	–	1.25(−0.80 to 3.29)	0.234	1905
Avoidance	Co-dominant	A/A	159	11.04(1.01)		0.603	1986
		A/G	83	12.69(1.34)	1.45(−1.62 to 4.51)		
		G/G	14	9.93(3.29)	−0.73(−7.06 to 5.61)		
	Dominant	A/A	159	11.04(1.01)		0.443	1984
		A/G–G/G	97	12.29(1.24)	1.14(−1.78 to 4.07)		
	Recessive	A/A–A/G	242	11.61(0.81)		0.693	1985
		G/G	14	9.93(3.29)	−1.26(−7.49 to 4.98)		
	Over-dominant	A/A–G/G	173	10.95(0.96)		0.326	1984
		A/G	83	12.69(1.34)	1.51(−1.50 to 4.52)		
	Log-additive	–	–	–	0.58(−1.81 to 2.97)	0.635	1985
Hyperarousal	Co-dominant	A/A	159	11.36(0.87)		0.407	1903
		A/G	83	13.06(1.15)	1.77(−0.84 to 4.38)		
		G/G	14	11.71(3.30)	1.12(−4.27 to 6.51)		
	Dominant	A/A	159	11.36(0.87)		0.186	1901
		A/G–G/G	97	12.87(1.09)	1.68(−0.80 to 4.17)		
	Recessive	A/A–A/G	242	11.94(0.70)		0.862	1903
		G/G	14	11.71(3.30)	0.47(−4.84 to 5.78)		
	Over-dominant	A/A–G/G	173	11.39(0.84)		0.201	1902
		A/G	83	13.06(1.15)	1.68(−0.89 to 4.24)		
	Log-additive	–	–	–	1.19(−0.84 to 3.22)	0.252	1902

*Cluster, PTSD symptom cluster; S.E, standard errors for each genotype; CI, confidence interval; AIC, Akaike information criterion of each genetic model;*

* *p < 0.05*.

## Discussion

The present study examined a genetic association between the *USP46* genetic variants and chronic PTSD among Korean male combat veterans. Single-marker analysis resulted in a nominally significant association only for rs2244291 with PTSD status, although the association did not remain significant after stringent correction for multiple comparisons. In addition, the rs2244291 was found to be associated with the “re-experiencing” cluster of PTSD symptoms. The present finding suggests preliminarily that some underlying genetic vulnerability regarding the ubiquitin-proteasome system such as USP46 may be related to fear memory processes and the development of some PTSD symptoms after trauma exposure.

To the best of our knowledge, the present study is the first to investigate the possible genetic association of the deubiquitinating enzyme in genetic susceptibility for PTSD. There is indirect evidence supporting the role of *USP46* in PTSD and fear memory processes. In animal studies, Ebihara and colleagues suggested that *Usp46* might be a quantitative trait gene responsible for immobility time reflecting behavioral despair under inescapable stress conditions (34). They showed that *Usp46* knockout mice exhibited shorter immobility times in the tail-suspension test, assessing depression-like behavior; reduced sucrose consumption in the sucrose preference test, assessing anhedonia-like symptoms; and lower locomotor activity levels in the open field test, assessing exploratory behavior and anxiety compared to wild type mice (33), which suggests the involvement of *Usp46* in stress-related phenotypes. In addition, ubiquitin-mediated protein degradation has been shown as important regulatory process in consolidation and extinction of memory in animal studies (23, 55, 56). Recent *in vitro* and *in vivo* findings showed that USP46 regulates glutamatergic receptor ubiquitination and turnover, as well as the strength of synaptic transmission, which suggest the involvement of *USP46* in synaptic plasticity and fear memory processes (29, 57). These findings are compatible with our finding that the *USP46* rs2244291 is associated with the “re-experiencing (having sudden and intrusive traumatic memories)” cluster, the core PTSD symptom, although fear memory processes themselves were not evaluated in the current study, when considering that re-experiencing of the traumatic event is closely related with abnormalities in fear memory processes including conditioning, reconsolidation and extinction of fear memory (58, 59). Particularly, rs2244291 has been reported to be involved in a haplotype pattern of susceptibility to major depression in a Japanese population by Fukuo et al. (44). Substantial genetic overlap between PTSD and depression has been reported (6, 60), which implies that genes implicated in the pathophysiology of depression are candidates for PTSD. When considering shared genetic components between PTSD and depression, genetic variations of ubiquitin-proteasome system, such as rs2244291, may be involved in regulating dynamic and reversible processes in synaptic plasticity and long-term potentiation after stress exposures in stress-related conditions, rather than in a disease-specific manner.

However, since no significant association with PTSD for the SNPs or the haplotype in the *USP46* region was detected after stringent correction for multiple comparisons in this Korean population, the present findings should be interpreted cautiously and preliminarily until confirmed. One possible reason for a weak association is that any one genetic polymorphism may confer a small genetic contribution to PTSD due to multi-factorial polygenic involvement in the pathophysiology of PTSD. The present negative findings in the main analysis should not be interpreted as conclusive for no association because the present sample might be too small for adequate statistical power to detect genetic variants with extremely small effect. Another possibility is that the weak association might be related to functional impact of other potential unmeasured genetic factors, such as the role of rare and structural genetic variations with strong effect. Although the role of rare and structural variation is not known in PTSD (61), rare variants were found to play unique roles in the genetics of complex diseases and research of rare variants require larger sample sizes than common variants to ensure sufficient statistical power (62). Further genetic studies in much larger samples will be helpful in unraveling the genetic contribution of common variants and rare variants to PTSD, its clusters, and broader phenotypes.

The strength of the present genetic association study is that case (trauma-exposed PTSD subjects)—control (trauma-exposed non-PTSD controls) design was applied for a relatively homogenous sample with exposure to similar trauma in a racially uniform population. However, limitations of this study should be noted. First, environmental factors such as early-life trauma were not controlled. Considering possible gene–environment interactions, some environments may have confounding effects that influence chronic PTSD status. Second, although our subjects are likely to comprise a more homogeneous sample with similar age and a single ethnic origin, the present study can only be regarded as a preliminary study in the Korean elderly population. Therefore, it should be replicated in larger sample sets, including populations with diverse ages and different ethnic backgrounds. Third, although we selected the *USP46* as a candidate gene based on a priori hypothesis on the possible role of the USP46 in synaptic plasticity and fear memory formation from previous studies, the functions of the present seven tag SNPs are unknown and no evidence of *USP46* has been reported from the GWAS studies on PTSD. Fourth, copy number variants or rare variants of *USP46* were not examined. In addition, gene expression analysis of the *USP46* was not conducted. Finally, psychiatric comorbidities such as depression might affect the present results. Since comorbidity of PTSD and depression could be largely explained by common genetic influences (60), we did not exclude the comorbidities. In the context of common genetic liability among PTSD, anxiety disorders, and depression, further research is required to determine genetic and environmental factors that influence the development of PTSD.

In summary, we investigated the clinical relevance of the genetic factors in the *USP46* using a case-control association design in Korean male veterans with or without PTSD after exposure to combat trauma. While the present findings suggest preliminarily that *USP46* rs2244291 may potentially be involved in re-experiencing symptoms and PTSD status after exposure to traumatic events, the limited sample size warrants caution for over-interpreting nominally significant genetic findings. Further research in large cohorts is needed to better understand the role of ubiquitin-proteasome system in genetic susceptibility to PTSD.

## Data Availability Statement

The raw data supporting the conclusions of this article will be made available by the authors, without undue reservation.

## Ethics Statement

The studies involving human participants were reviewed and approved by institutional review board of the VHS Medical Center, South Korea (BOHUN 2016-02-007). The patients/participants provided their written informed consent to participate in this study.

## Author Contributions

JK and TK designed the study. TK, JC, and HS collected the data. JS, JK, SK, and HS undertook the statistical analyses and interpreted the findings. JS and JK prepared the manuscript. All authors contributed to, and approved, the final manuscript.

## Conflict of Interest

The authors declare that the research was conducted in the absence of any commercial or financial relationships that could be construed as a potential conflict of interest.

## Publisher's Note

All claims expressed in this article are solely those of the authors and do not necessarily represent those of their affiliated organizations, or those of the publisher, the editors and the reviewers. Any product that may be evaluated in this article, or claim that may be made by its manufacturer, is not guaranteed or endorsed by the publisher.

## Abbreviations

USP46: ubiquitin carboxyl-terminal hydrolase 46
PTSD: post-traumatic stress disorder
USP: ubiquitin specific peptidase
GABA: gamma-aminobutyric acid
AMPA: alpha-amino-3-hydroxy-5-methyl-4-isoxazolepropionic acid
VHS: veterans health service
CAPS: clinician-administered PTSD scale
CES: combat exposure scale
AUDIT: alcohol use disorders identification test
SNPs: single nucleotide polymorphisms
LD: linkage disequilibrium.
