# Supplementary material for: Possible Association of Polymorphisms in Ubiquitin Specific Peptidase 46 Gene With Post-traumatic Stress Disorder
Source: Front Psychiatry. 2021 Aug 11;12:663647. doi: 10.3389/fpsyt.2021.663647 (PMC8385240; doi:10.3389/fpsyt.2021.663647)
Supplement: Supplementary file 1 [file Data_Sheet_1.docx]

**Table S1. Primer sequences used in the analysis of *USP46* SNPs**

| **Gene** | **rs number** | **Strand** | **Primer sequence** | |
| --- | --- | --- | --- | --- |
| *USP46* | rs346005 | Forward | Forward Primer | GAAAGCTCTTCAGCACACA |
|  |  |  | Reverse Primer | AAACAAGGTCAAAGCAGTTT |
|  |  |  | Genotyping Primer | GCAAGGGCTCGAGACACAGACA |
|  | rs10034164 | Forward | Forward Primer | CCAAATAAGAAGGCAGCTT |
|  |  |  | Reverse Primer | CACAGTCCCAAGAAGGTCT |
|  |  |  | Genotyping Primer | GGCCTCAGTGAAGCCTACAGTAAC |
|  | rs2244291 | Forward | Forward Primer | CAAAAGCTCTCAACCTTCC |
|  |  |  | Reverse Primer | TGTCATCTGCACAAACAGA |
|  |  |  | Genotyping Primer | AGAACAATTGCACAGCCTATTTTT |
|  | rs12646800 | Forward | Forward Primer | AACATCTCCAGTGCTCTCC |
|  |  |  | Reverse Primer | TGTCTGTTTGTTGGAAGGA |
|  |  |  | Genotyping Primer | agtgagcctggcaYgcttggtgtcc |
|  | rs6554557 | Forward | Forward Primer | Taacacgatcttggctcac |
|  |  |  | Reverse Primer | ggaaaagttcaagccaatc |
|  |  |  | Genotyping Primer | ttRcatacgtgagccacAGTKTGCAGT |
|  | rs17675844 | Forward | Forward Primer | ttggtgaggaccttctttc |
|  |  |  | Reverse Primer | CTCTGGGTGGGAAGATTAC |
|  |  |  | Genotyping Primer | CATCAACATCTATGTAAAGGG |
|  | rs10517263 | Forward | Forward Primer | ctggcactacaggtttgtg |
|  |  |  | Reverse Primer | CCCCACTAAGGGTCAGTTA |
|  |  |  | Genotyping Primer | CTTATTCATTTAAAAATACCTGTCT |

**Table S2. Characteristics of SNP markers on the *USP46* gene in the non-PTSD group and PTSD group**

| rs number | Location ^a^ | Non-PTSD group | | | | PTSD group | | | |
| --- | --- | --- | --- | --- | --- | --- | --- | --- | --- |
|  |  | χ^2^ | *p* value ^b^ | Allele | MAF | χ^2^ | *p* value ^b^ | Allele | MAF |
| rs346005 | 52588572 | 2.509 | 0.113 | A>C | 0.432 | 0.392 | 0.531 | A>C | 0.440 |
| rs10034164 | 52593565 | 0.008 | 0.929 | T>C | 0.156 | 2.577 | 0.108 | T>C | 0.153 |
| rs2244291 | 52602313 | 3.776 | 0.076 | A>G | 0.198 | 0.699 | 0.403 | A>G | 0.237 |
| rs12646800 | 52602534 | 0.336 | 0.562 | C>T | 0.050 | 0.624 | 0.430 | C>T | 0.068 |
| rs6554557 | 52621374 | 0.008 | 0.929 | A>C | 0.156 | 1.010 | 0.315 | A>C | 0.150 |
| rs17675844 | 52624583 | 0.276 | 0.599 | A>C | 0.073 | 0.003 | 0.955 | A>C | 0.096 |
| rs10517263 | 52632502 | 0.165 | 0.685 | C>G | 0.110 | 0.001 | 0.977 | C>G | 0.093 |

SNP, single nucleotide polymorphism; MAF, minor allele frequency

^a^ Information on the chromosomal position is based on NCBI genome build GRCh37.p13.

^b^ *P* value for Hardy-Weinberg equilibrium
